# Clinical, cytogenetic, and molecular findings of isodicentric Y chromosomes

**DOI:** 10.1186/s13039-019-0465-x

**Published:** 2019-12-27

**Authors:** Yang Yang, Wang Hao

**Affiliations:** 1 Prenatal Diagnosis Center, Hangzhou Maternity and Child Care Hospital, #369 Kunpeng Road, Shangcheng District, Hangzhou, 310008 Zhejiang China; 2 Department of Cell Biology and Medical Genetics, School of Medicine, Zhejiang University, Hangzhou, Zhejiang China

**Keywords:** Isodicentric Y chromosome, Fluorescence in situ hybridization, Chromosomal microarray analysis, Prenatal diagnosis, Mosaicism

## Abstract

**Background:**

Isodicentric Y chromosomes [idic(Y)] are one of the most common structural abnormalities of the Y chromosome. The prenatal diagnosis of isodicentric Y chromosomes is of vital importance, and the postnatal phenotypes vary widely. Therefore, we present six patients prenatally diagnosed with isodicentric Y chromosomes and review the literature concerning the genotype-phenotype correlations.

**Method:**

The clinical materials of six patients were obtained. Cytogenetic and molecular approaches were carried out for these six patients.

**Results:**

Isodicentric Y chromosomes were found in all sixpatients. Among them, four patients presented with a mosaic 45,X karyotype, one patient had a 46,XY cell line, and one patient was nonmosaic. Five of these six isodicentric Y chromosomes had a breakpoint in Yq11.2, and the other had a breakpoint in Yp11.3. The molecular analysis demonstrated different duplications and deletions of the Y chromosome. Finally, three patients chose to terminate the pregnancy, two patients gave birth to normal-appearing males, and one patient was lost to follow-up.

**Conclusion:**

The incorporation of multiple cytogenetic and molecular techniques would offer a more comprehensive understanding of this structural chromosomal abnormality for genetic counselling.

## Background

Isodicentric Y chromosomes [idic(Y)] were first identified by Jacobs et al. [1] and are commonly found in Y chromosome structural aberrations [2]. The formation of isodicentric Y chromosomes is believed to result from intrachromosomal recombination or the fusion between sister chromatids following the chromosomal break of the Y chromosome [3]. The sites where breakage and fusion occur at the Y chromosome vary greatly [4]. Thus, the isodicentric Y chromosome breakpoint would determine the Y material maintained, leading to highly variable duplications and deletions of the Y chromosome.

Isodicentric Y chromosomes are quite unstable due to the existence of two centromeres, resulting in various mosaicism [5]. The karyotypes of the mosaic cell lines depend on the origin where the isodicentric Y chromosomes arose and the instability of the altered chromosomes during meiosis or mitosis [6], and a 45,X cell line is the most common [7]. Patients with isodicentric Y chromosomes have a wide range of phenotypic manifestations, such as Turner syndrome in females [8], infertility in males [9], ambiguous genitalia [10], gonadal dysgenesis [11], short stature [12] and others. The phenotypes depend on the breakage and fusion of the isodicentric Y chromosomes, as well as the types and proportions of mosaicism [13].

Given that isodicentric Y chromosomes have great impacts on the genotypes and phenotypes of affected patients, a prenatal diagnosis is crucial. Several approaches, including cytogenetic and molecular techniques, are generally applied for the prenatal diagnosis of isodicentric Y chromosomes. The methods can be complementary to each other due to their own merits and limitations.

Here, we present six patients with isodicentric Y chromosomes identified prenatally using different detection approaches to emphasize the importance of combining conventional cytogenetic analyses with molecular techniques in prenatal diagnosis. Furthermore, we reviewed the relevant literature aiming to better understand the genotype-phenotype correlations of isodicentric Y chromosomes for comprehensive genetic counselling.

## Methods

### Subjects

Six patients were referred to the Prenatal Diagnosis Centre of our hospital for various indications. Amniotic fluid samples were obtained from all patients, and cord blood was obtained from patient 6. All patients were informed of the test they were to complete. The clinical information of the patients is listed in Table 1.

**Table 1:** Clinical information of six patients with isodicentric Y chromosomes

Patient	Age (years)	Indication	Pregnancy history	Specimen	Laboratory test
1	36	AMA	G2 P1	AF	Karyotype, BoBs assay, FISH
2	34	AMA	G2 P1	AF	Karyotype, FISH
3	28	NIPT abnormality	G2 P1	AF	Karyotype, CMA, FISH
4	35	NIPT abnormality	G2 P1	AF	Karyotype, CMA
5	30	MSS and NIPT abnormality	G2 P0	AF	Karyotype, CMA
6	40	AMA, IVF	G3 P1	AF, CB	Karyotype, FISH

*AMA* Advanced maternal age, *NIPT* Non-invasive prenatal test, *MSS* Maternal serum screening, *IVF* In vitro fertilization, *AF* Amniotic fluid, *CB* Cord blood, *BoBs* Bacterial artificial chromosomes-on-beads, *FISH* Fluorescence in situ hybridization, *CMA* Chromosomal microarray analysis

### Cytogenetic analysis

Amniotic fluid and cord blood samples were obtained via transabdominal amniocentesis and cordocentesis under sterile circumstances. Amniotic fluid cells and cord blood lymphocytes were cultured and harvested according to standard protocols. G-band staining was applied for the preparation of the chromosome specimens. The karyotypes were reported in accordance with the up-to-date International System for Human Cytogenomics Nomenclature 2016 (ISCN2016).

### Fluorescence in situ hybridization (FISH) analysis

FISH analysis was carried out using a sex-determining region Y (SRY)/CEP X (DXZ1) probe and a CEP Y (DYZ3) probe (Vysis; Abbott Molecular, IL, USA) following the manufacturer’s instructions. The SRY/DXZ1 probe specifically hybridizes with the sex-determining region of Yp11.3 and the X centromere. The DYZ3 probe is specific for the centromeric region of the Y chromosome.

### Bacterial artificial chromosomes-on-beads assay

The prenatal bacterial artificial chromosomes-on-beads (BoBs) assay was performed according to the manufacturer’s protocol (PerkinElmer, MA, USA). The kit was designed for the aneuploidies of chromosomes 13, 18, and 21 and the sex chromosomes, as well as the detection of 9 microdeletion syndromes. The beads were analysed using the Luminex 200 platform (Luminex,TX, USA), and the data analysis was performed with BoBsoft 2.0 software (PerkinElmer, MA, USA).

### Chromosomal microarray analysis (CMA)

The Affymetrix CytoScan 750 K gene chip (Affymetrix, CA, USA) was used for the CMA. It contains 200,000 single nucleotide polymorphism (SNP) probes and 550,000 copy number variant (CNV) probes. The data were analysed using Chromosome Analysis Suite software (Affymetrix, CA, USA), and changes in CNVs, loss of heterozygosity (LOH) and uniparental disomy (UPD) were identified.

## Results

**Fig. 1:**
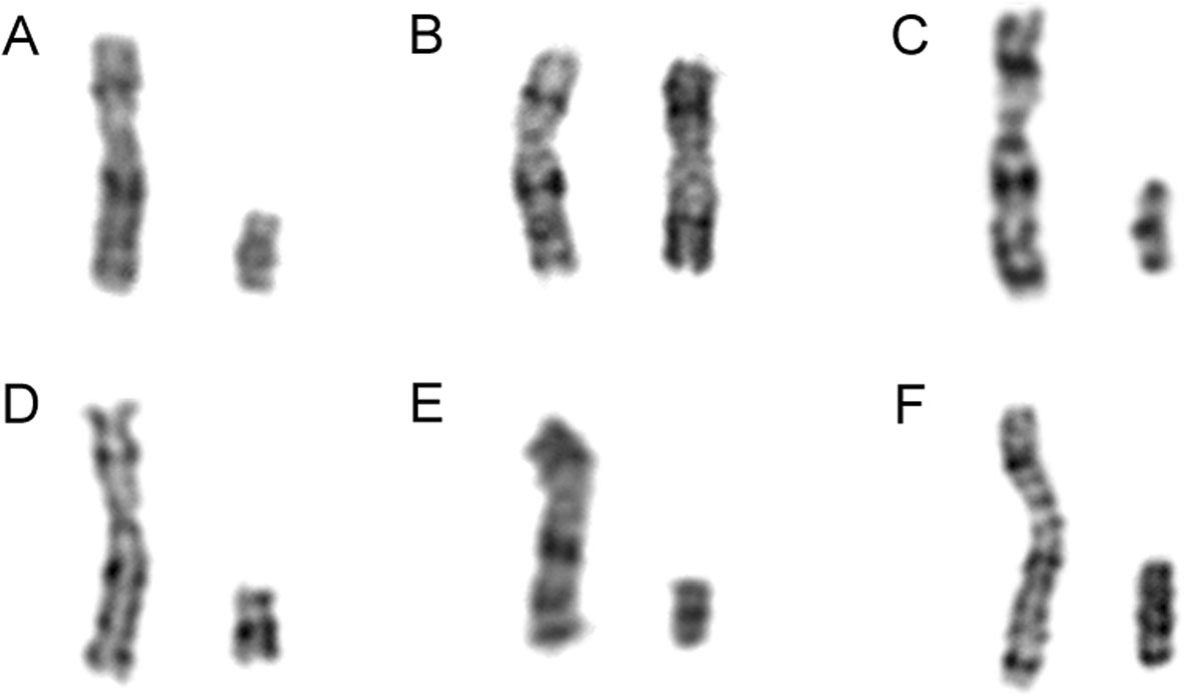
Partial karyotypes of X and Y chromosomes for the six patients. All the Y chromosomes are isodicentric and placed on the right. **a** Patient 1: idic(Y) (q11.21). **b** Patient 2: idic(Y) (p11.3). **c** Patient 3: idic(Y)(q11.2). **d** Patient 4: idic(Y)(q11.22). **e** Patient 5: idic(Y) (q11.2). **f** Patient 6: idic(Y) (q11.2)

**Fig. 2:**
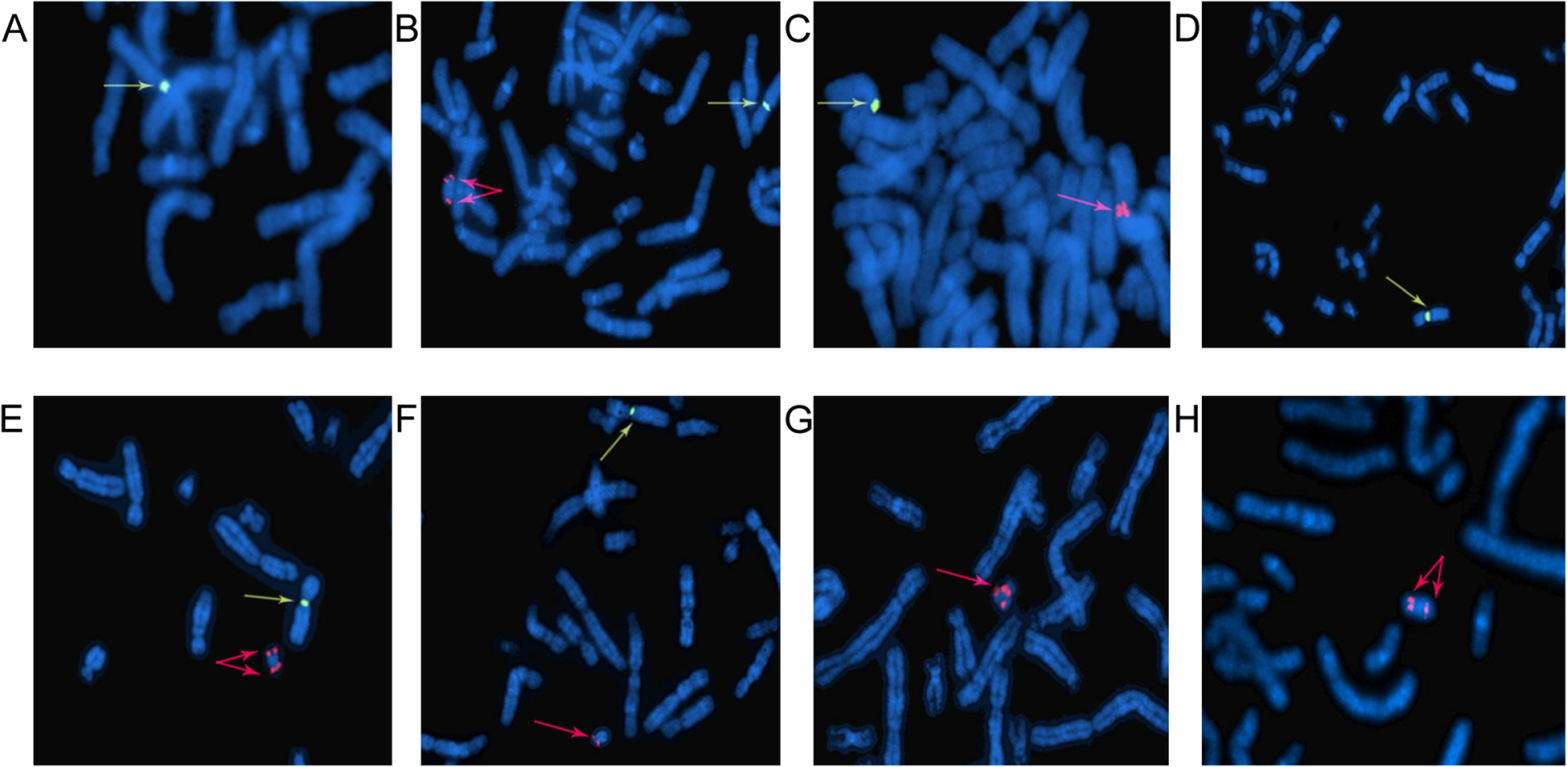
FISH analysis for patients 1, 2, 3, and 6. Patient 1: the SRY/DXZ1 probe was used. **a** Only one DXZ1 (green) signal was observed, indicating a 45,X karyotype. **b** Two SRY (red) signals and one DXZ1 (green) signal were observed, suggesting a 46,X,idic(Y) karyotype. Patient 2: the SRY/DXZ1 probe was used. **c** Two proximal SRY signals are shown in red, revealing that the breakpoint of this isodicentric Y chromosome was in the short arm of the Y chromosome. Patient 3: The different patterns of SRY (red) and DXZ1 (green) signals in (**d**), (**e**), and (**f**) suggested mosaicism of 45,X/46,X,idic(Y)/46,XY. **g** The DYZ3 probe was also applied to patient 3. One DYZ3 (red) centromeric signal was constricted while the other one was not, indicating that one centromere was inactivated. Patient 6: **h** Two DYZ3 (red) signals were observed at the isodicentric Y chromosome

The cytogenetic analysis of cultured amniotic fluid samples from patient 1 revealed a 45,X [32]/46,X,idic(Y)(q11.21) [1] karyotype (Fig. 1a). FISH analysis showed two SRY signals in 7 of 100 uncultured amniocytes, and no SRY signal was found in the remaining cells (Fig. 2a, b). The prenatal BoBs assay indicated microdeletions in the Yq11.223 region, which was consistent with the breakpoint in Yq11.2. Ultrasound examination indicated male genitalia. After a great deal of deliberation, the patient decided to terminate the pregnancy.

The karyotype of cultured amniocytes obtained from patient 2 was 46,X,?idic(Y) (Fig. 1b). FISH analysis of uncultured amniocytes confirmed the presence of an isodicentric Y chromosome with the breakpoint at Yp11.3 in all 50 cells counted (Fig. 2c). The SRY signals were closelylocated. Finally, the karyotype of the foetus was reported as 46,X,idic(Y)(p11.3).ish idic(Y)(p11.3)(SRY++). The patient decided to continue the pregnancy and gave birth to a phenotypically normal male.

The evaluation of G-banding chromosomes of amniocytes from patient 3 showed a mosaic karyotype of 46,X,idic(Y)(q11.2) [59]/45,X [14] (Fig. 1c). FISH analysis revealed that 42% of the uncultured amniocytes had a 45,X karyotype, 38% of the counted cells contained an isodicentric Y chromosome, and 20% of the cells showed a normal male karyotype (Fig. 2d, e, f, g). The CMA of the amniotic fluid sample suggested a 19.4 Mb duplication of the Yp11.32q11.221 segment and a 38.7 Mb deletion of the Yq11.222q12 segment. This finding confirmed the breakpoint in Yq11.2. The peripheral blood of the foetus’s father was also obtained, and no abnormality of the Y chromosome was found in the karyotype analysis. Termination of the pregnancy was performed after genetic counselling.

Regarding patient 4, the karyotype of cultured amniocytes was mos 45,X [17]/46,X, idic(Y)(q11.22) [9] (Fig. 1d). The CMA of the amniotic fluid specimen indicated a 17.9 Mb duplication of the Yp11.31q11.222 segment and a 7.7 Mb deletion of the Yq11.222q11.23 segment. The pregnancy was continued, and the infant was an apparently normal male at birth.

The karyotype of cultured amniocytes of patient 5 was mos 45,X [26]/46,X,idic(Y)(q11.2) [5] (Fig. 1e). A 16.9 Mb duplication of the Yp11.31q11.221 segment and an 8.1 Mb deletion of the Yq11.222q11.23 segment were found in the CMA of the amniotic fluid. The patient chose to terminate the pregnancy based on the results.

The karyotypes of amniotic fluid and cord blood from patient 6 were 46,X,idic(Y)(q11.2) [17]/46,XY [15] and 46,X,idic(Y)(q11.2), respectively. This isodicentric Y chromosome is displayed in Fig. 1f. FISH analysis was applied to the cultured suspension of cord blood to confirm that the derivative Y chromosome had two visible centromeres (Fig. 2h). Patient 6 was lost to follow-up, and thus, the clinical outcome for her foetus was not obtained.

## Discussion

Isodicentric Y chromosomes are frequently observed in Y chromosome structural abnormalities [2]. This Y chromosome aberration involves the breakage and fusion of the Y chromosome, which leads to the gain and loss of Y chromosome material. As the Y chromosome contains various genes involved in sex determination, spermatogenesis, growth and development, deletions and duplications of the Y chromosome would probably cause multiple malformations and dysfunctions in the affected individual. Thus, it is extremely important to identify this Y chromosome aberration prenatally to provide genetic counselling and interpretation. Previous studies have applied conventional karyotype analysis, FISH, Southern blot, and sequence-tagged site (STS) PCR to identify isodicentric Y chromosomes and the breakpoints contained within them [14, 15]. Other techniques, such as multiplex ligation-dependent probe amplification (MLPA), qPCR, CMA, and the sequencing of certain genes, have also been used to gain molecular insight into isodicentric Y chromosomes [16–18]. In this study, we used different methods for the detection of isodicentric Y chromosomes and found that they could be complementary to each other. Because of the limitation of each technique, using one single method to identify isodicentric Y chromosomes could be quite risky, especially when it coexists with other cell lines. A combination of cytogenetic and molecular analysis would provide detailed information on the gain and loss of isodicentric Y chromosomes, assisting in the interpretations of the test results [19]. However, inconsistent results might arise in cases of very complicated mosaicism, which require apanoramic view of the results in a retrospective manner [17]. Additionally, cryptic mosaics make it even more difficult to identify small supernumerary marker chromosomes [20]. Researchers have established pericentromeric-critical region FISH probe sets to better characterize small supernumerary marker chromosomes that are also applied to isodicentric Y chromosomes [21].

Because of the existence of two centromeres in isodicentric Y chromosomes, it is difficult for this derivative chromosome to remain stable. Researchers have noted that isodicentric Y chromosomes can achieve stabilization via the inactivation of one centromere, and the active centromere, which is constricted, would bind to the mitotic spindles [22]. This is consistent with the findings of our study. Nonetheless, isodicentric Y chromosomes appear to be either monocentric ordicentric depending on the intercentromeric distance [22]. If the intercentromeric distance is small enough, the two active centromeres could behave as one centromere [23]. In some cases, isodicentric Y chromosomes with one or two constrictions could coexist in one specimen [24]. Given the instability of isodicentric Y chromosomes, they frequently appear as highly mosaic [23]. In our study, only one of the six patients had a nonmosaic karyotype, and a 45,X cell line was most common form of mosaicism. These findings are consistent with most isodicentric Y chromosome cases reported [4, 11, 14, 25–29]. There are also many other aberrant chromosomes that could arise with isodicentric Y chromosomes, leading to a very complicated karyotype [3, 4, 17, 30–32]. This mosaicism might be related to the time (i.e., during meiosis or postzygote) during which the isodicentric Y chromosome originated, instability during mitosis, and whether other chromosomes were involved [23]. Dynamic mosaicism could make the karyotypes even more variable [32].

Patients carrying isodicentric Y chromosomes often come to medical attention when certain abnormal manifestations emerge. The prenatal diagnosis of isodicentric Y chromosomes is less common than the postnatal diagnosis of isodicentric Y chromosomes [33]. The prenatally diagnosed foetuses with isodicentric Y chromosomes are listed in Table 2. Most were phenotypical males, even carrying a 45,X cell line. 45,X cell lines predominated in the amniotic fluid karyotypes of the two females but presented lower proportions in the cord blood [34, 36]. A discrepancy between mosaicism from different tissues has generally been recognized in other cases [9, 11, 18, 43–45], which might have been caused by the different origins of the tissues and could be affected by the biases of subcultures and counting. Therefore, we could not determine the accurate percentage of a 45,X cell line to indicate the phenotypical sex of the foetuses. Even if the foetus was male, the percentage of a 45,X cell line could be rather high, as we observed in patient 1. The low percentage of isodicentric Y chromosomes in patient 1 could have been missed if it were not for the ultrasound result. Thus, the ultrasound determination of the phenotypical sex of the foetuses could aid in the cytogenetic analysis [36]. The patients described in Table 2 and our patients showed that most isodicentric Y chromosomes found prenatally had similar breakpoints in Yq11.2 and Yp11.3, but the breakpoints had no direct correlation with the phenotypes at birth or termination. As listed in Table 2, some foetuses presented normal phenotypes, while others showed various defects because of the gain and loss of genetic material. Considering that the follow-up was not longterm, abnormalities might arise during puberty. Clinical management will be very important along with the growth and development of the affected individuals.

**Table 2:** Summary of patients prenatally diagnosed with isodicentric Y chromosomes

Patient	Tissue	Karyotype	Clinical outcome	Sex	Reference
1	AF	47,X,idic(Y)(q11.21),inv. dup(Y)	Phenotypically normal	M	[3]
	PB				
2	AF	46,X,idic(Y)(q11.23)	Phenotypically normal	M	[23]
3	AF	46,X,del(Y)(q12)/45,X/46,X,idic(Y)(q11.22)	Unknown	M	[17]
4	AF	46,X,idic(Y)(q11.23) [41]/45,X [22]	Phenotypically normal	M	[5]
5	AF	45,X [54]/46,X,idic(Y)(p11.3) [8]/46,XY [3]	Defect in the interventricular septum of the heart	M	[18]
	Antenatal CB	45,X [24]/46,X,idic(Y)(p11.3) [26]			
	Placental villi	45,X[89]/46,X,idic(Y)(p11.3) [11]			
	Postnatal CB	45,X[87]/46,X,idic(Y)(p11.3) [10]/46,XY [3]			
	Gonad	45,X [45]/46,X,idic(Y)(p11.3) [55]			
6	AF	45,X	Termination	F	[34]
	CB	45,X(20%)/46,X,idic(Y)(p11)(80%)			
7	AF	45,X [27]/46,X,idic(Y)(q11.22) [14]	Termination	M	[19]
8	PB	45,X [23]/46,X,idic(Y) [8]	Complex heart lesion, generalized oedema, died 19 days after birth	A	[35]
	Right Gonad	45,X [47]/46,X,idic(Y) [3]			
	Left Gonad	45,X [44]/46,X,idic(Y) [6]			
	AF	45,X			
9	AF	45,X [28]/46,X,idic(Y)(q11.2) [2]	Phenotypically normal	F	[36]
	CB	46,X,idic(Y)(q11.2) [31]/45,X [17]/47,X,idic(Y)× 2 [2]			
10	AF	46,X,idic(Y) [3]/45,X [2]/46,XY [24]	Phenotypically normal	M	[25]
11	AF	45,X [2]/46,XY [13]	Phenotypically normal	M	[25]
	PB	46,X,idic(Y)(q10) [5]/46,XY[95]			
12	Chorionic villi	45,X [11]/46,XY [9]	Phenotypically normal	M	[25]
	AF	46,X,idic(Y)(q11) [4]/45,X [1]/46,XY [26]			
13	AF	46,X,idic(Y)(q10) [26]/45,X [3]/47,X,i(Y),+i(Y) [2]	Phenotypically normal	M	[25]
	PB	46,X,idic(Y)(q11)[95]/45,X [5]			
14	AF	46,X,idic(Y)(q11.2) [17]	Phenotypically normal	M	[25]
	PB	46,X,idic(Y)(q11.2) [50]			
	Chorion	46,X,idic(Y)(q11.2) [27]/45,X [3]			
15	AF	45,X/46,X,idic(Y)(q11.2)/47,X,idic(Y)(q11.2),+idic(Y)(q11.2)	Phenotypically normal	M	[25]
	PB	46,X,idic(Y)(q11.2) [18]/45,X [14]			
16	AF	46,idic(Y)(q11.2) [13]/45,X [6]	Phenotypically normal	M	[25]
	PB	46,X,idic(Y)(q11.2) [8]/45,X [2]			
17	AF	46,X,idic(Y)(q11.2) [22]/45,X [7]	Normal genitalia at termination	M	[25]
	Chorion	46,X,idic(Y)(q11.1) [8]			
18	AF	46,X,idic(Y) [22]/45,X [5]	Phenotypically normal	M	[25]
19	AF	45,X [12]/46,XY [17]	Phenotypically normal	M	[25]
	Amnion	45,X [2]/46,XY [11]			
	PB	46,X,idic(Y)(q11.2) [17]/45,X [3]/46,X,?r(Y) [2]			
20	AF	45,X [10]	Normal genitalia at termination	M	[25]
	Villi, amnion	45,X/46,X,idic(Y)(q11.2)			
21	AF	45,X [10]	Ambiguous genitalia, short stature	A	[25]
	PB	45,X/46,X,idic(Y)(q11.2)			
	Skin	45,X/46,X,idic(Y)(q11.2)/46,X,?r(Y)			
22	AF	45,X, [14]/46,X,psu dic(Y)(q12) [5]	Phenotypically normal	M	[27]
23	AF	45,X [3]/46,X,idic(Y)(p11) [11]	Phenotypically normal	M	[27]
24	AF	45,X [2]/46,X,idic(Yp) [14]	Normal genitalia at 9 years, coarctation of the aorta	M	[27]
25	PB	46,X,idic(Y)(q11.21)	Mild language delay	M	[7]
	AF	46,X,idic(Y)(q11.21)			
26	AF	45,X [14]/46,X,idic(Y)(q11.2)[86]	Unilateral renal agenesis, normal genitalia	M	[37]
27	AF	45,X [15]/46,X,idic(Yp) [6]/46,X,?del(Y)(q12) [2]/47,X,?del(Y)(q12) +?del(Y)(q12) [2]	Termination	–	[38]
	Foetal heart	45,X [12]/46,X,idic(Yp) [9]/46,X,?del(Y)(q12) [4]			
	Foetal kidney	45,X [19]/46,X,idic(Yp) [3]/46,X,?del(Y)(q12) [2]/47,X,?del(Y)(q12) +?del(Y)(q12) [1]			
28	AF	45,X/46,X,idic(Y)	Phenotypically normal	M	[39]
29	AF	45,X/46,X,idic(Y)	Phenotypically normal	M	[39]
30	AF	45,X[125]/46,X,dic(Y)(q11) [5]	Abdominal wall defect, mild chordee of the penis	M	[40]
	PB	45,X [14]/46,X,dic(Y)(q11) [16]			
	Skin	45,X [15]/46,X,dic(Y)(q11) [15]			
	Placenta A	45,X [4]/46,X,dic(Y)(q11) [30]			
	Placenta B	45,X [20]/46,X,dic(Y)(q11) [10]			
	Placenta C	45,X [12]/46,X,dic(Y)(q11) [18]			
31	AF	45,X [8]/46,X,idic(Y)(p11.32) [2]	Phenotypically normal	M	[41]
	PB	45,X [27]/46,X,idic(Y) [1]			
32	PB	45,X/46,X,idic(Y)(q11.2)	Unknown	F	[42]

*AF* Amniotic fluid, *CB* Cord blood, *PB* Peripheral blood, *M* Male, *F* Female, *A* Ambiguous

The most common clinical manifestations of patients with isodicentric Y chromosomes are stigmata of Turner syndrome, gonadal dysgenesis, ambiguous genitalia, growth and mental retardation, azoospermia, infertility and others [4, 8–12, 16, 28–30, 35, 41, 42]. The phenotypes are related to the breakpoints of isodicentric Y chromosomes, mosaicism, and distributions of cell lines in different tissues [13]. Isodicentric Y chromosomes would lose segments from the breakpoints to the distal ends and gain partial disomy of the segments maintained. Y chromosomes are more likely to break in common fragile AT-rich sites [46]. Patients with isodicentric Y chromosomes showing symptoms of Turner syndrome often carry a 45,X cell line [4]. The SRY gene is located at Yp11.32 and is critical for the development of secondary sexual characteristics in males [47]. Yp11.32 is a very common breakpoint in isodicentric Y chromosomes [48–51]. However, the copy number of the SRY gene could not determine the phenotype of the patient due to the coexistence of other cell lines. Some patients with two copies of the SRY gene on isodicentric Y chromosomes still had ambiguous genitalia resulting from mosaicism [11]. The azoospermia and infertility observed in patients with isodicentric Y chromosomes were primarily associated with breakpoints in Yq, leading to deletions and rearrangements of azoospermia factor (AZF) loci (AZFa, AZFb, and AZFc) [52]. These three loci are all involved in spermatogenesis, and the loss of any locus would cause oligozoospermia or azoospermia [53]. However, some researchers reported that a patient without AZF deletions demonstrated azoospermia possibly due to other Y chromosome structural abnormalities or mosaicism [54]. Yp11.32 also contains the short stature homeobox (SHOX) gene, which participates in the proliferation and differentiation of chondrocytes [55] and hence growth retardation in affected patients. Some patients showed features of Klinefelter syndrome resulting from extra Y chromosome material [31, 56, 57]. A few researchers found a potential correlation between isodicentric Y chromosomes and susceptibility to schizophrenia [58], but the evidence was not strong enough [59]. There are some other rare defects that occur in patients carrying isodicentric Y chromosomes, such as Moyamoya disease, aortic dissection, and congenital heart disease [18, 35, 60, 61]. These are either coincidences or consequences of the altered dosage of sex chromosome genes [60, 61]. Individual differences in development also play a vital role in the clinical manifestations of patients [62–64]. In general, it is still difficult to conclude a precise genotype-phenotype relationship.

Once isodicentric Y chromosomes are identified in affected patients, medical interventions should be proposed. Patients with a short stature could achieve near-adult height with growth hormone therapy from an early age [33]. Female patients carrying isodicentric Y material in the gonads are at great risk of gonadoblastoma, especially after puberty; thus, prophylactic gonadectomy is strongly recommended [65–67]. Foetuses showing ambiguous genitalia should be assigned a certain sex after a thorough evaluation of the genital anomalies [68]. Because patients carrying isodicentric Y chromosomes often present with complex manifestations, a long-term follow-up and clinical management are of great importance.

In conclusion, we reported six patients prenatally diagnosed with isodicentric Y chromosomes using cytogenetic and molecular techniques. Because isodicentric Y chromosomes often present with mosaicism, we need to be careful when addressing these cases. The application of multiple methods to identify isodicentric Y chromosomes could not only serve as confirmation but also provide more detailed information of the derivative chromosomes for genetic counselling. Clinical information such as ultrasound results could help uncover low mosaics of isodicentric Y chromosomes. A long-term follow-up would help shed light on the genotype-phenotype relationship of isodicentric Y chromosomes.

## Data Availability

The datasets used and/or analysed during the current study are available from the corresponding author on reasonable request.
